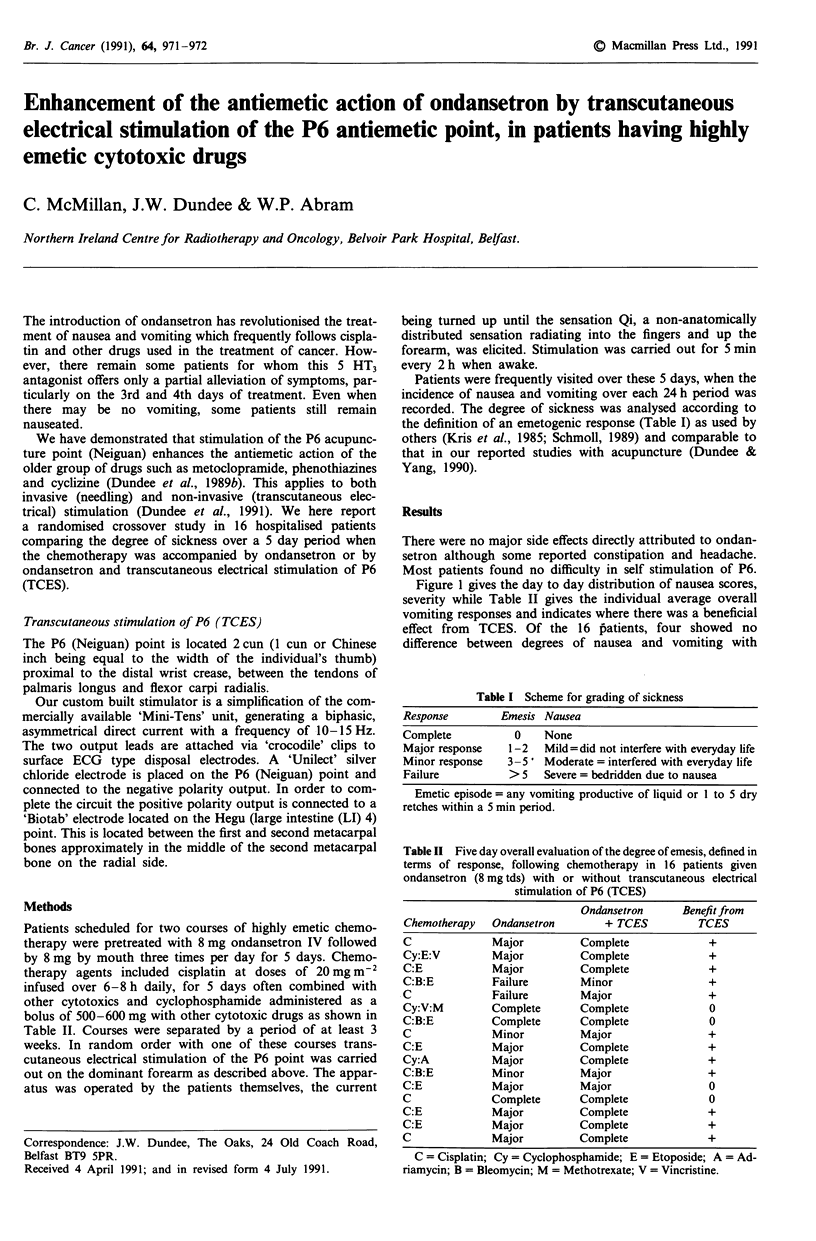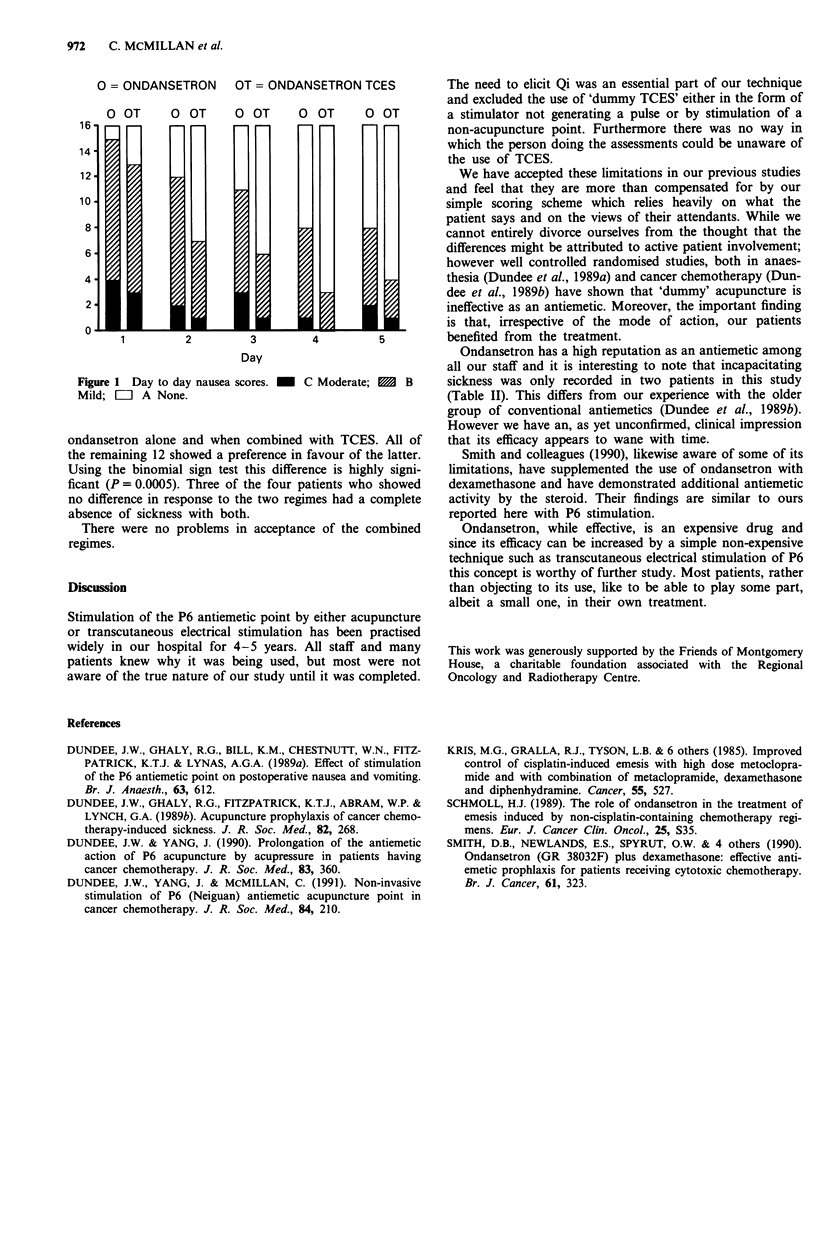# Enhancement of the antiemetic action of ondansetron by transcutaneous electrical stimulation of the P6 antiemetic point, in patients having highly emetic cytotoxic drugs.

**DOI:** 10.1038/bjc.1991.439

**Published:** 1991-11

**Authors:** C. McMillan, J. W. Dundee, W. P. Abram

**Affiliations:** Northern Ireland Centre for Radiotherapy and Oncology, Belvoir Park Hospital, Belfast.


Br. J. Cancer (1991), 64, 971–972 © Macmillan Press Ltd., 1991

### Enhancement of the antiemetic action of ondansetron by transcutaneous electrical stimulation of the P6 antiemetic point, in patients having highly emetic cytotoxic drugs

C. McMillan, J.W. Dundee & W.P. Abram

*Northern Ireland Centre for Radiotherapy and Oncology, Belvoir Park Hospital, Belfast.*

The introduction of ondansetron has revolutionised the treatment of nausea and vomiting which frequently follows cisplatin and other drugs used in the treatment of cancer. However, there remain some patients for whom this 5 $HT_3$ antagonist offers only a partial alleviation of symptoms, particularly on the 3rd and 4th days of treatment. Even when there may be no vomiting, some patients still remain nauseated.

We have demonstrated that stimulation of the P6 acupuncture point (Neiguan) enhances the antiemetic action of the older group of drugs such as metoclopramide, phenothiazines and cyclizine (Dundee *et al*., 1989*b*). This applies to both invasive (needling) and non-invasive (transcutaneous electrical) stimulation (Dundee *et al*., 1991). We here report a randomised crossover study in 16 hospitalised patients comparing the degree of sickness over a 5 day period when the chemotherapy was accompanied by ondansetron or by ondansetron and transcutaneous electrical stimulation of P6 (TCES).

*Transcutaneous stimulation of P6 (TCES)*

The P6 (Neiguan) point is located 2 cun (1 cun or Chinese inch being equal to the width of the individual's thumb) proximal to the distal wrist crease, between the tendons of palmaris longus and flexor carpi radialis.

Our custom built stimulator is a simplification of the commercially available 'Mini-Tens' unit, generating a biphasic, asymmetrical direct current with a frequency of 10–15 Hz. The two output leads are attached via 'crocodile' clips to surface ECG type disposal electrodes. A 'Unilect' silver chloride electrode is placed on the P6 (Neiguan) point and connected to the negative polarity output. In order to complete the circuit the positive polarity output is connected to a 'Biotab' electrode located on the Hegu (large intestine (LI) 4) point. This is located between the first and second metacarpal bones approximately in the middle of the second metacarpal bone on the radial side.

### Methods

Patients scheduled for two courses of highly emetic chemotherapy were pretreated with 8 mg ondansetron IV followed by 8 mg by mouth three times per day for 5 days. Chemotherapy agents included cisplatin at doses of 20 mg $m^{-2}$ infused over 6–8 h daily, for 5 days often combined with other cytotoxics and cyclophosphamide administered as a bolus of 500–600 mg with other cytotoxic drugs as shown in Table II. Courses were separated by a period of at least 3 weeks. In random order with one of these courses transcutaneous electrical stimulation of the P6 point was carried out on the dominant forearm as described above. The apparatus was operated by the patients themselves, the current being turned up until the sensation Qi, a non-anatomically distributed sensation radiating into the fingers and up the forearm, was elicited. Stimulation was carried out for 5 min every 2 h when awake.

Patients were frequently visited over these 5 days, when the incidence of nausea and vomiting over each 24 h period was recorded. The degree of sickness was analysed according to the definition of an emetogenic response (Table I) as used by others (Kris *et al*., 1985; Schmoll, 1989) and comparable to that in our reported studies with acupuncture (Dundee & Yang, 1990).

### Results

There were no major side effects directly attributed to ondansetron although some reported constipation and headache. Most patients found no difficulty in self stimulation of P6.

Figure 1 gives the day to day distribution of nausea scores, severity while Table II gives the individual average overall vomiting responses and indicates where there was a beneficial effect from TCES. Of the 16 patients, four showed no difference between degrees of nausea and vomiting with

**Table I** Scheme for grading of sickness

| *Response* | *Emesis* | *Nausea* |
|---|---|---|
| Complete | 0 | None |
| Major response | 1–2 | Mild = did not interfere with everyday life |
| Minor response | 3–5 | Moderate = interfered with everyday life |
| Failure | >5 | Severe = bedridden due to nausea |

Emetic episode = any vomiting productive of liquid or 1 to 5 dry retches within a 5 min period.

**Table II** Five day overall evaluation of the degree of emesis, defined in terms of response, following chemotherapy in 16 patients given ondansetron (8 mg tds) with or without transcutaneous electrical stimulation of P6 (TCES)

| *Chemotherapy* | *Ondansetron* | *Ondansetron + TCES* | *Benefit from TCES* |
|---|---|---|---|
| C | Major | Complete | + |
| Cy:E:V | Major | Complete | + |
| C:E | Major | Complete | + |
| C:B:E | Failure | Minor | + |
| C | Failure | Major | + |
| Cy:V:M | Complete | Complete | 0 |
| C:B:E | Complete | Complete | 0 |
| C | Minor | Major | + |
| C:E | Major | Complete | + |
| Cy:A | Major | Complete | + |
| C:B:E | Minor | Major | + |
| C:E | Major | Major | 0 |
| C | Complete | Complete | 0 |
| C:E | Major | Complete | + |
| C:E | Major | Complete | + |
| C | Major | Complete | + |

C = Cisplatin; Cy = Cyclophosphamide; E = Etoposide; A = Adriamycin; B = Bleomycin; M = Methotrexate; V = Vincristine.

Correspondence: J.W. Dundee, The Oaks, 24 Old Coach Road, Belfast BT9 5PR.
Received 4 April 1991; and in revised form 4 July 1991.

**Figure 1** Day to day nausea scores. ■ C Moderate; ▨ B Mild; □ A None.

ondansetron alone and when combined with TCES. All of the remaining 12 showed a preference in favour of the latter. Using the binomial sign test this difference is highly significant ($P = 0.0005$). Three of the four patients who showed no difference in response to the two regimes had a complete absence of sickness with both.

There were no problems in acceptance of the combined regimes.

### Discussion

Stimulation of the P6 antiemetic point by either acupuncture or transcutaneous electrical stimulation has been practised widely in our hospital for 4–5 years. All staff and many patients knew why it was being used, but most were not aware of the true nature of our study until it was completed. The need to elicit Qi was an essential part of our technique and excluded the use of 'dummy TCES' either in the form of a stimulator not generating a pulse or by stimulation of a non-acupuncture point. Furthermore there was no way in which the person doing the assessments could be unaware of the use of TCES.

We have accepted these limitations in our previous studies and feel that they are more than compensated for by our simple scoring scheme which relies heavily on what the patient says and on the views of their attendants. While we cannot entirely divorce ourselves from the thought that the differences might be attributed to active patient involvement; however well controlled randomised studies, both in anaesthesia (Dundee *et al.*, 1989*a*) and cancer chemotherapy (Dundee *et al.*, 1989*b*) have shown that 'dummy' acupuncture is ineffective as an antiemetic. Moreover, the important finding is that, irrespective of the mode of action, our patients benefited from the treatment.

Ondansetron has a high reputation as an antiemetic among all our staff and it is interesting to note that incapacitating sickness was only recorded in two patients in this study (Table II). This differs from our experience with the older group of conventional antiemetics (Dundee *et al.*, 1989*b*). However we have an, as yet unconfirmed, clinical impression that its efficacy appears to wane with time.

Smith and colleagues (1990), likewise aware of some of its limitations, have supplemented the use of ondansetron with dexamethasone and have demonstrated additional antiemetic activity by the steroid. Their findings are similar to ours reported here with P6 stimulation.

Ondansetron, while effective, is an expensive drug and since its efficacy can be increased by a simple non-expensive technique such as transcutaneous electrical stimulation of P6 this concept is worthy of further study. Most patients, rather than objecting to its use, like to be able to play some part, albeit a small one, in their own treatment.

This work was generously supported by the Friends of Montgomery House, a charitable foundation associated with the Regional Oncology and Radiotherapy Centre.

### References

DUNDEE, J.W., GHALY, R.G., BILL, K.M., CHESTNUTT, W.N., FITZPATRICK, K.T.J. & LYNAS, A.G.A. (1989*a*). Effect of stimulation of the P6 antiemetic point on postoperative nausea and vomiting. *Br. J. Anaesth.*, **63,** 612.

DUNDEE, J.W., GHALY, R.G., FITZPATRICK, K.T.J., ABRAM, W.P. & LYNCH, G.A. (1989*b*). Acupuncture prophylaxis of cancer chemotherapy-induced sickness. *J. R. Soc. Med.*, **82,** 268.

DUNDEE, J.W. & YANG, J. (1990). Prolongation of the antiemetic action of P6 acupuncture by acupressure in patients having cancer chemotherapy. *J. R. Soc. Med.*, **83,** 360.

DUNDEE, J.W., YANG, J. & MCMILLAN, C. (1991). Non-invasive stimulation of P6 (Neiguan) antiemetic acupuncture point in cancer chemotherapy. *J. R. Soc. Med.*, **84,** 210.

KRIS, M.G., GRALLA, R.J., TYSON, L.B. & 6 others (1985). Improved control of cisplatin-induced emesis with high dose metoclopramide and with combination of metaclopramide, dexamethasone and diphenhydramine. *Cancer*, **55,** 527.

SCHMOLL, H.J. (1989). The role of ondansetron in the treatment of emesis induced by non-cisplatin-containing chemotherapy regimens. *Eur. J. Cancer Clin. Oncol.*, **25,** S35.

SMITH, D.B., NEWLANDS, E.S., SPYRUT, O.W. & 4 others (1990). Ondansetron (GR 38032F) plus dexamethasone: effective antiemetic prophlaxis for patients receiving cytotoxic chemotherapy. *Br. J. Cancer*, **61,** 323.